# Predictors of Quality of Life in Acromegaly: No Consensus on Biochemical Parameters

**DOI:** 10.3389/fendo.2017.00040

**Published:** 2017-03-03

**Authors:** Victor J. Geraedts, Cornelie D. Andela, Günter K. Stalla, Alberto M. Pereira, Wouter R. van Furth, Caroline Sievers, Nienke R. Biermasz

**Affiliations:** ^1^Department of Clinical Neuroendocrinology, Max Planck Institut für Psychiatrie, Munich, Germany; ^2^Department of Medicine, Division of Endocrinology, Leiden University Medical Center, Leiden, Netherlands; ^3^Department of Medicine, Division of Neurosurgery, Leiden University Medical Center, Leiden, Netherlands

**Keywords:** acromegaly, quality of life, systematic review, depression, biochemical control

## Abstract

**Background:**

Quality of life (QoL) in patients with acromegaly is reduced irrespective of disease state. The contributions of multifactorial determinants of QoL in several disease stages are presently not well known.

**Objective:**

To systematically review predictors of QoL in acromegalic patients.

**Methods:**

Main databases were systematically searched using predefined search terms for potentially relevant articles up to January 2017. Inclusion criteria included separate acromegaly cohort, non-hereditary acromegaly, QoL as study parameter with clearly described method of measurement and quantitative results, *N* ≥ 10 patients, article in English and adult patients only. Data extraction was performed by two independent reviewers; studies were included using the PRISMA flow diagram.

**Results:**

We identified 1,162 studies; 51 studies met the inclusion criteria: 31 cross-sectional observational studies [mean AcroQoL score 62.7 (range 46.6–87.0, *n* = 1,597)], 9 had a longitudinal component [mean baseline AcroQoL score 61.4 (range 54.3–69.0, *n* = 386)], and 15 were intervention studies [mean baseline AcroQoL score 58.6 (range 52.2–75.3, *n* = 521)]. Disease-activity reflected by biochemical control measures yielded mixed, and therefore inconclusive results with respect to their effect on QoL. Addition of pegvisomant to somatostatin analogs and start of lanreotide autogel resulted in improvement in QoL. Data from intervention studies on other treatment modalities were too limited to draw conclusions on the effects of these modalities on QoL. Interestingly, higher BMI and greater degree of depression showed consistently negative associations with QoL. Hypopituitarism was not significantly correlated with QoL in acromegaly.

**Conclusion:**

At present, there is insufficient published data to support that biochemical control, or treatment of acromegaly in general, is associated with improved QoL. Studies with somatostatin receptor ligand treatment, i.e., particularly lanreotide autogel and pegvisomant have shown improved QoL, but consensus on the correlation with biochemical control is missing. Longitudinal studies investigating predictors in treatment-naive patients and their follow-up after therapeutic interventions are lacking but are urgently needed. Other factors, i.e., depression and obesity were identified from cross-sectional cohort studies as consistent factors associated with poor QoL. Perhaps treatment strategies of acromegaly patients should not only focus on normalizing biochemical markers but emphasize improvement of QoL by alternative interventions such as psychosocial or weight lowering interventions.

## Introduction

**Figure 1:**
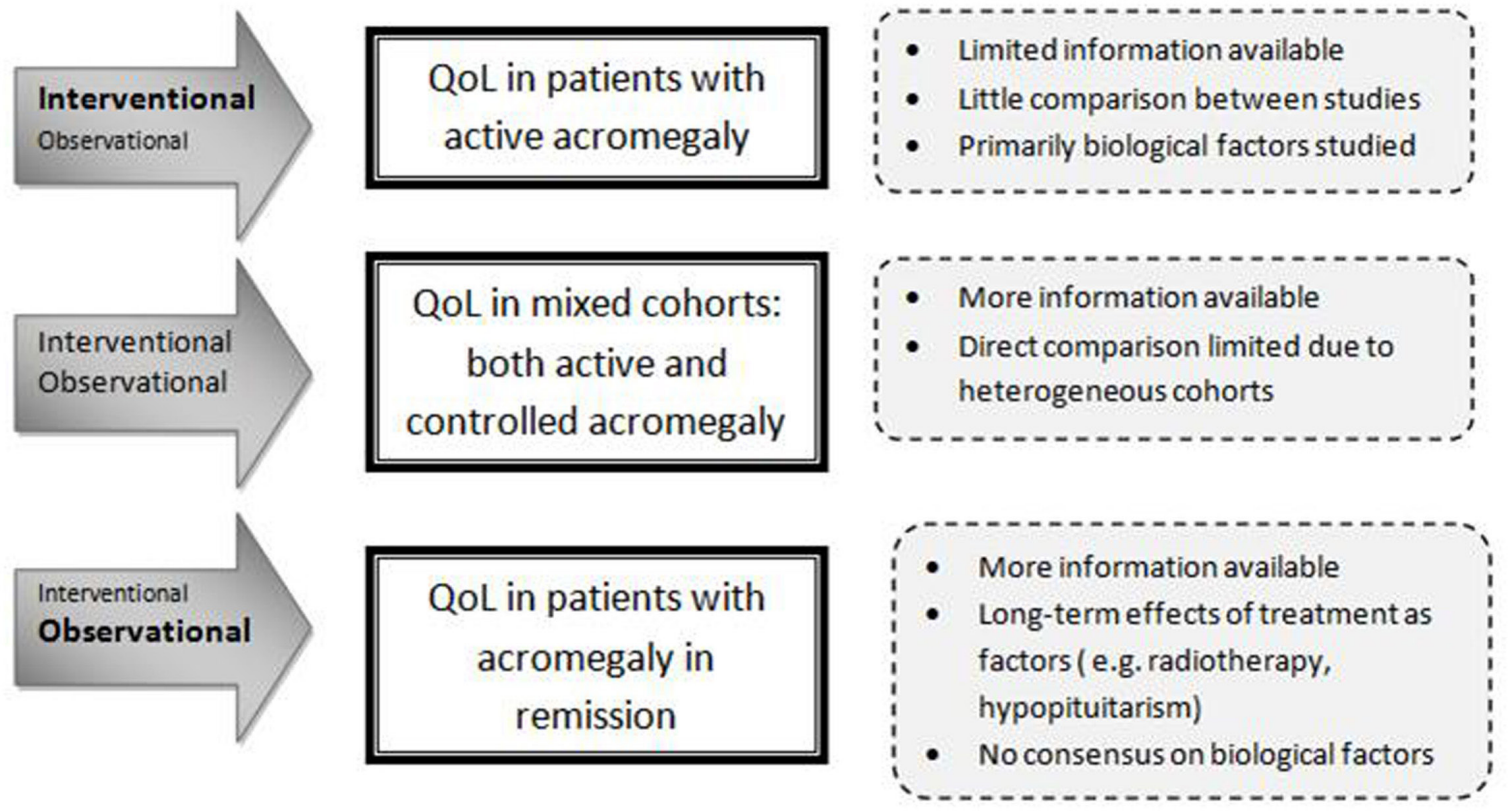
**Interpretation of the setting of the studied predictors**.

The World Health Organization recognizes three patient-related health outcome goals in chronic disease management: reducing mortality, reducing morbidity, and improving quality of life (QoL) (1). QoL is a multidimensional entity that represents the functional effect of an illness and its consequent therapy upon a patient, as perceived by the patient (2). The initial focus on reducing mortality and improving morbidity as well as normalizing biochemical target values, i.e., IGF-I and growth hormone (GH), in patients with acromegaly has yielded successful results (3–5). Nevertheless, QoL remains a major concern, since it often remains reduced despite long-term biochemical cure (6). In both patients with active or controlled acromegaly, QoL has been reported to be markedly decreased relative to the normal population, with some improvement after treatment. Studies, usually cross-sectional designed because of the rarity of the disease, have been exploring disease-related and general factors (e.g., age, gender) that can affect QoL of patients with acromegaly. These studies included rather heterogeneous groups of patients with acromegaly (in terms of disease stages, extent of disease control, and treatment history) (7, 8). Literature reports conflicting results about factors that affect QoL. For example, a number of studies found no correlation between biochemical control of acromegaly and QoL (9–11), whereas others reported a significant correlation (12, 13). A more consistent finding during long-term follow-up of acromegaly patients is the high prevalence of joint complaints, fatigue, and (neuro)psychological problems. There is increasing evidence that in patients with acromegaly an initial period of GH excess can cause permanent complications, despite long-term cure. This has been shown, e.g., with structural changes in macroscopic brain architecture, irreversible radiological joint abnormalities, and body composition (14–18). As QoL in the general sense is a multifactorial entity, it is plausible to assume that QoL in patients with acromegaly is also determined by several factors, which may differ depending on the phase of the disease. Factors that may be of importance during the untreated phase of the disease (i.e., active disease) are not necessarily of equal importance during the early treatment phase (i.e., transition from active to controlled disease) and the subsequent phase of acromegaly in chronic-treated situation (usually remission). It is important to acknowledge different study designs and the timing of the QoL measurement in relation to the disease phase, and lack of available data when analyzing factors associated with QoL in acromegaly. For example, interventional studies focus predominantly on active or treatment-naïve patients. A limited number of longitudinal observational studies have included patients with changing disease status. Long-term effects of treatment, such as post-radiation effects or hypopituitarism, are inherent to treated acromegaly and will have a more prominent role than in active acromegaly. Cohorts including patients with both active and controlled disease are therefore particularly heterogeneous, limiting direct comparison (see also Figure 1). Based on evidence based medicine, it is crucial to identify which factors are most influential on QoL during a certain phase of the disease in order to develop suitable interventions aimed at improving QoL. Therefore, the aim of this systematic literature study was to evaluate predictors of QoL in patients with acromegaly in several stages of their diseases and to identify potentially modifiable factors as targets for interventions.

## Methods

This systematic review aimed to adhere to the current PRISMA guidelines (19).

### Data Sources and Search

Seven electronic databases were searched for potentially relevant articles. PubMed, EMBASE, Web of Science, PsycINFO, Academic Search Premier, COCHRANE, and CENTRAL were searched using the keywords “Pituitary Neoplasms,” “Pituitary Neoplasm,” “Pituitary Tumors,” “Pituitary Tumor,” “Pituitary Tumours,” “Pituitary Tumour,” “Pituitary Adenomas,” “Pituitary Adenoma,” “Growth Hormone-Secreting Pituitary Adenoma,” “Growth Hormone-Secreting Adenomas,” “Acromegaly,” “Quality of Life,” “Life quality,” “qol,” “daily functioning,” “daily routine,” health related QoL,” “well-being,” and “wellbeing.”

### Study Selection

Articles were retrieved based on analysis of title and abstract whether they met the following six inclusion criteria: (1) separate acromegaly cohort, (2) non-hereditary acromegaly, (3) QoL as parameter, with clearly described method of measurement and quantitative results, (4) *N* ≥ 10 patients, (5) article in English, and (6) adult patients only.

Articles detailing cohorts detailing growth hormone deficiency (GHD) after acromegaly were considered a separate entity and excluded from the analysis. Studies based on similar cohorts were separately included.

### Data Extraction and Risk of Bias Assessment

Screening of potentially suitable articles, as well as assessing eligibility, was performed by two independent reviewers. Inclusion in the final systematic review was done upon mutual agreement.

Information about the study size, origin of the patients, outcome, and potential conflicts of interest were extracted from each study.

Only factors that were described in more than one article were included for the systematic review part. Factors were scored having (1) no significant association with QoL, (2) a significant association with a subscale of the QoL-instrument (positive- or negative association), or (3) a significant association with the total QoL-instrument (positive- or negative association). Results were stratified into general factors, disease-specific factors, or interventions inherently linked to acromegaly. Consensus was determined as all studies unidirectionally described.

Quality of the selected articles was assessed using the Newcastle-Ottawa Quality Assessment Scale (NOS) for cohort studies/case control studies (20). The maximum score for each article was 4 stars for the item “selection,” 2 stars for the item “comparability,” and 3 stars for either the item “outcome” or “exposure,” respectively.

A customized evaluation tool (see Table 1) was drafted by our research group and used to determine the quality of QoL assessment specifically, with a maximum of 10 points. This tool comprises biological, psychological, and social elements of a patients’ QoL and further differentiates between generic and disease-specific QoL, allowing for both a global QoL assessment and a specific design (e.g., AcroQoL).

**Table 1:** **Quality of life (QoL) quality assessment (max. 10 points)**.

Item	Scoring method
QoL primary outcome	No = 0
	Yes = 1
Group composition	Heterogeneous: active/controlled = 0
	Homogenous: active vs./or controlled = 1
Questionnaires	Generic only = 0
	Disease-specific = 0
	Domain-specific = 0
	Generic + disease-specific = 1
	Generic + domain-specific = 1
	Disease-specific + domain-specific = 1
	Generic + disease-specific + domain-specific = 2
Validation questionnaires	No = 0
	Yes, general validation = 1
	Yes, validated in cultural study population = 2
QoL domains assessed	One domain BPS = 0
	Two domains BPS = 1
	Three domains BPS = 2
*Bio-psycho-social*	Three domains BPS + other = 3
Discussion of clinical implication QoL scores	No = 0
	Yes = 1

## Results

### Search Results and Study Characteristics

**Figure 2:**
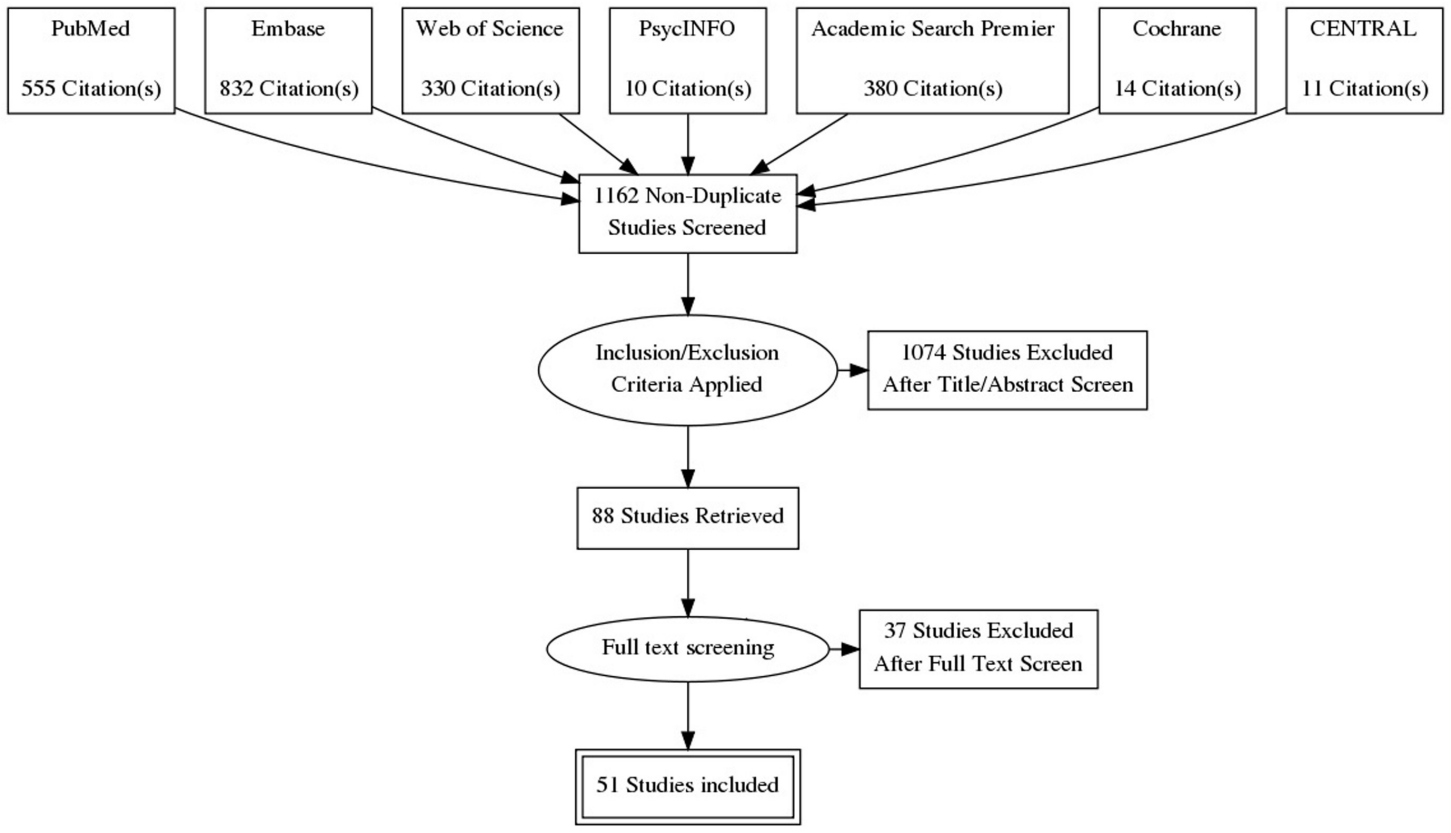
**Prisma flow diagram**.

The search yielded 1,162 articles, of which 1,074 were excluded based on the title and abstract. The 88 remaining studies were checked for the aforementioned inclusion criteria; 15 of these studies were excluded on the basis of no description of predictors of QoL, and another 9 studies because the patients had been diagnosed with GHD after treatment for acromegaly. Fifty-one studies were ultimately included (see also Figure 2): 8 case–control studies and 43 cohort studies. Naturally, for the case–control studies, only the cohorts of patients with acromegaly were studied in this review. Thirty-one studies were classified as cross-sectional observational studies, 9 had an observational longitudinal component, and 15 studies were intervention studies. The selected studies, as well as abstracted data, are shown in Table 2.

**Table 2:** **Selected studies**.

Reference	Study type	Country, region of origin cohort	*N*	QoL Questionnaires
Anagnostis et al. (21)	Case–control^a^	Greece, Thessaloniki	40	AcroQoL

Biermasz et al. (22)	Cohort, intervention	The Netherlands, Leiden	14	NHP

Biermasz et al. (23)	Case–control^a^	The Netherlands, Leiden	118	AcroQoL, SF36, NHP, MFI-20, HADS

Biermasz et al. (24)	Cohort	The Netherlands, Leiden	118	AcroQoL, SF36, NHP, MFI-20, HADS

Bonapart et al. (25)	Cohort, longitudinal	The Netherlands, Rotterdam	14	SF36

Bronstein et al. (26)	Cohort, longitudinal	Brazil, multicenter trial	119	AcroQoL

Cannavo et al. (27)	Case–control^a^	Italy Messina	56	AcroQoL

Caron et al. (28)	Cohort, intervention	France, multicenter trial: 27 centers in 9 countries	90	AcroQoL

Caron et al. (29)	Cohort, intervention	France, multicenter trial: 27 centers in 9 countries	90	AcroQoL

Celik et al. (30)	Cohort	Turkey, Istanbul	57	AcroQoL

Celik and Kadioglu (31)	Cohort	Turkey, Istanbul	57	AcroQoL

Chin et al. (32),	Cohort, intervention	Korea, multicenter trial: 11 centers in Korea	58	AcroQoL

Dantas et al. (33)	Cohort	Brazil, Brasilia	42	SF36

Fathalla et al. (34)	Cohort	Canada, Toronto	20	SF36, PIT QOL

Fujio et al. (35)	Cohort, intervention	Japan, Kagoshima	41	SF36

Geraedts et al. (36)	Cohort, longitudinal	Germany, Munich	80	AcroQoL, SF36

Ghigo et al. (37)	Cohort, intervention	Italy, multicenter trial: 50 centers in 13 countries	113	AcroQoL

Hatipoglu et al. (38)	Case–control, intervention^a^	Turkey, Istanbul	20	AcroQoL

Hatipoglu et al. (39)	Cohort	Turkey, Istanbul	30	AcroQoL

Hua et al. (9)	Cohort	Taiwan, Taipei	52	AcroQoL

Karaca et al. (40)	Cohort, intervention	Turkey, Kayseri	22	AcroQoL

Kauppinen-Makelin et al. (41)	Cohort	Finland, multicenter trial: 5 centers in Finland	231	15D

Kepicoglu et al. (42)	Cohort	Turkey, Istanbul	133	AcroQoL

Leon-Carrion et al. (43)	Case–control^a^	Spain, multicenter trial: 4 centers in Spain	34	AcroQoL

Lombardi et al. (44)	Cohort, intervention	Italy, multicenter trial: 24 centers in Italy	16	NHP

Madsen et al. (45)	Cohort, intervention	Denmark, Aarhus	51	EuroQoL

Mangupli et al. (46)	Cohort, intervention	Venezuela, Caracas	18	AcroQoL

Matta et al. (47)	Cohort	France, Toulouse	28	AcroQoL

Milian et al. (48)	Cohort, intervention	Germany, Tuebingen	93	AcroQoL, SF36, QLS-H, SCL-90

Miller et al. (49)	Cohort	United Kingdom, Oxford	58	AcroQoL, SF36, AIMS2

Neggers et al. (50)	Cohort, intervention	The Netherlands, Rotterdam	30	AcroQoL

Paisley et al. (12)	Cohort, longidutinal	United Kingdom, Manchester	56	AcroQoL, EUroQoL, PGWB, SSS

Postma et al. (51)	Cohort	The Netherlands, multicenter trial: 2 centers in The Netherlands	108	AcroQoL, SF36, MFI-20, HADS

Psaras et al. (52)	Cohort	Germany, Tuebingen	37	AcroQoL, SF36, SCL-90-R

Psaras et al. (53)	Cohort	Germany, Tuebingen	55	AcroQoL, SF36

Raappana et al. (54)	Cohort	Finland, Oulou	22	15D

Roerink et al. (55)	Case–control^a^	The Netherlands, Nijmegen	73	AcroQoL, SF36

Rowles et al. (56)	Cohort	United Kingdom, Manchester	80	AcroQoL, EuroQoL, PGWB, SSS

Rubeck et al. (57)	Cohort	Denmark, Aarhus	63	EuroQoL

Sardella et al. (58)	Cohort, longitudinal	Italy, Pisa	23	AcroQoL

Schopohl et al. (59),	Cohort	Germany, multicenter trial: 13 centers in Germany	17	AcroQoL

Siegel et al. (60)	Cohort	Germany, Aachen	41	AcroQoL, SF36

T’Sjoen et al. (10)	Cohort	Belgium, multicenter trial: 37 centers in Belgium and Luxembourg	291	AcroQoL

Trainer et al. (61)	Cohort, intervention	United Kingdom, multicenter trial: 29 centers	77	AcroQoL, EuroQoL

Trepp et al. (13)	Cohort	Switzerland, Bern	33	AcroQoL

van der Klaauw et al. (62)	Cohort, longitudinal	The Netherlands, Leiden	82	AcroQoL, SF36, HADS, MFI-20

Vandeva et al. (63)	Cohort, longitudinal	Bulgaria, Sofia	212	AcroQoL

Varewijck et al. (64)	Cohort	The Netherlands, Rotterdam	15	AcroQoL, SF36

Wassenaar et al. (65)	Cohort	The Netherlands, Leiden	58	AcroQoL, SF36, MFI-20, HADS

Webb et al. (66)	Case–control, longitudinal^a^	Spain, multicenter trial: 16 centers in Spain	106	AcroQoL, EuroQoL

Yoshida et al. (67)	Cohort	Japan, Kobe	38	AcroQoL

*^a^For case–control studies, only the cohorts detailing patients with acromegaly were studied*.

### Baseline AcroQol-Scores

Eighty percent of studies used the disease-specific measurement instrument AcroQoL (*n* = 41). Mean AcroQoL scores in cross-sectional studies were 62.7 (range 46.6–87.0, *n* = 1597, maximum score = 100). Baseline mean AcroQoL scores in longitudinal studies were of 61.4 (range 54.3–69.0, *n* = 386), and 58.6 (range 52.2–75.3, *n* = 521) in intervention studies. Given the heterogeneity of the individual studies, no formal conclusions can be drawn as to whether the means of the different study types are statistically different.

### Quality Assessment

The quality assessment with regard to general quality (NOS) and specific QoL quality can be found in Table S1 in Supplementary Material. Five studies were classified as high quality studies (NOS ≥ 8), 33 studies were classified as medium quality studies (NOS 6-7), and 13 studies were classified as low quality studies (NOS ≤ 5). Quality of QoL assessment was high in 12 studies (≥9 points), medium in 25 studies (6–8 points), and low in 14 studies (≤5 points).

### Described Factors/Interventions

Disease-specific factors that were identified from cross-sectional and longitudinal studies were biochemical control (*n* = 23), IGF1 (*n* = 17), GH (*n* = 11), hypopituitarism (*n* = 10), disease duration (*n* = 8), tumor size (*n* = 5), duration of remission (*n* = 4), nadir GH (*n* = 3), change in IGF1 (*n* = 2), and follow-up duration (*n* = 2). General predictors that were identified were age (*n* = 16), gender (*n* = 14), depression scores (*n* = 7), education (*n* = 5), BMI (*n* = 2), and physical activity (*n* = 2). (Retrospective) Interventions identified from cross-sectional studies were a history of radiotherapy (*n* = 15), surgery (*n* = 8), current use of somatostatin analogs (*n* = 4), and application of any treatment of acromegaly (not otherwise specified) (*n* = 2).

Interventions identified from intervention trials (QoL measured in patients with acromegaly before and after intervention) were somatostatin analogs (*n* = 8), pegvisomant (*n* = 3), and pituitary surgery (*n* = 3).

Results indicating whether a factor was described to have a significant positive, significant negative, or no significant effect are denoted in Table 3 for general and disease-specific factors, and in Table 4 for applied interventions (therapies).

**Table 3:** **Factors influencing QoL in acromegaly from cross-sectional observational studies**.

Reference	General factors					Disease-specific factors							
	Age	Female gender	Depression	Education	BMI	Biochemical control	Hypopituitarism	GH	Disease duration	IGF1	Tumor size	Remission duration	Followup duration
Anagnostis et al. (21)	0	−−	−−	0		0	0	0^a^			0	0	
Biermasz et al. (23)	−	0	−−				0	+	0	0		−	−
Biermasz et al. (24)	−−							+	−−				
Cannavo et al. (27)								0		−−			
Celik et al. (30)						0							
Celik and Kadioglu (31)			−−	++		0	0						
Dantas et al. (33)						+							
Fathalla et al. (34)						0					0		
Hatipoglu et al. (38)			−−							−−			
Hatipoglu et al. (39)						0	0	0	0	0			
Hua et al. (9)						0							
Karaca et al. (40)						0							
Kauppinen-Makelin et al. (41)	−−	0			−−		0	0, −−^a^		−−	++		
Kepicoglu et al. (42)	0	0	−−	++		−−	0		0	++			
Leon-Carrion et al. (43)			−−					++		++			
Mangupli et al. (46)								0		0			
Matta et al. (47)						+							
Milian et al. (48)		−−		0		0							0
Miller et al. (49)									0				
Postma et al. (51)	0	−−								0			
Psaras et al. (52)	0	0				−				0			
Psaras et al. (53)	0	0				0		0		0	0		
Raappana et al. (54)						0							
Rowles et al. (56)	0	0					0		0				
Siegel et al. (60)										−−			
T’Sjoen et al. (10)	0	−−			−−	0					0		
Trepp et al. (13)						++				−−			
Vandeva et al. (63)	−−	−−				0							
Varewijck et al. (64)										−			
Wassenaar et al. (65)	+/−						0	0, 0^a^	0	0		0	
Yoshida et al. (67)	−−					0				−−			

*++ positive correlation with QoL, +positive correlation with a subscale of QoL only, −−negative correlation with QoL, −negative correlation with a subscale of QoL only, 0 no significant correlation with QoL*.

*^a^Nadir growth hormone (GH)*.

**Table 4:** **Factors influencing QoL in acromegaly in cross-sectional observational studies (previous and ongoing interventions)**.

Reference	Treatment of acromegaly	Radiotherapy	Somatostatin analogs	Surgery without adjuvant treatment	Pituitary surgery	Physical activity	Use of GH-lowering medication	Number of surgeries	Surgery vs. somatostatin analogs	Lanreotide autogel after treatment with octreotide
Anagnostis et al. (21)	0	−−								
Biermasz et al. (23)		−	0	+						
Biermasz et al. (24)		−								
Celik and Kadioglu (31)					++					
Dantas et al. (33)						0				
Fathalla et al. (34)		−−	−−					0		
Hatipoglu et al. (39)		0			0					
Kauppinen-Makelin et al. (41)		−−			0		0			
Kepicoglu et al. (42)		−−						0		
Matta et al. (47)					0					
Postma et al. (51)		0								
Raappana et al. (54)			−−							
Rowles et al. (56)		−−								
Rubeck et al. (57)									0	
Schopohl et al. (59)										0
T’Sjoen et al. (10)	0	0								
Vandeva et al. (63)		−−	0					−−		
Wassenaar et al. (65)		−							0	
Yoshida et al. (67)		−−							+	

*++positive correlation with QoL, +positive correlation with a subscale of QoL only, −− negative correlation with QoL, −negative correlation with a subscale of QoL only, 0 no significant correlation with QoL*.

### Results from Cross-sectional Studies Irrespective of Disease Status

In heterogeneous cohorts with both active and controlled disease, general factors that had a negative effect on QoL in patients with acromegaly were higher depression scores (21, 23, 31, 38, 42, 43) and higher BMI (10, 41) (see also Tables 3 and 4). The disease-specific factor hypopituitarism was described to have no significant effect (39, 41, 56, 65), while previous/ongoing treatments could not be associated with QoL in acromegaly [i.e., any treatment of acromegaly (not otherwise specified) (10, 21), surgery vs. somatostatin analogs (57, 65)] and physical activity (33). Other predictors were either not reported, or no consensus was reached on other predictors, such as the demographic factors age and gender, the biochemical parameters GH, IGF1, or biochemical control, and the duration of either disease or remission.

### Results from Cross-sectional Studies Stratified for Disease Status

In cohorts with patients with active acromegaly only (six studies), the general factors depression scores (43), age and female gender (63), had a significant negative effect on QoL. The disease-specific factor GH level was described to be positively correlated with QoL (43) (see Table 5). Other predictors were either not reported, or no consensus was reached between the respective articles, such as IGF1, biochemical control, and disease duration.

**Table 5:** **Factors influencing QoL in patients with active acromegaly (cross-sectional)**.

Reference	General factors			Disease-specific factors			
	Depression	Age	Female gender	IGF1	GH	Disease duration	Biochemical control
Hua et al. (9)							0
Leon-Carrion et al. (43)	−−			++	++		
Matta et al. (47)							+
Sardella et al. (58)						0	−
Vandeva et al. (63)		−−	−−	0			
Varewijck et al. (64)				−			

*++positive correlation with QoL, +positive correlation with a subscale of QoL only, −−negative correlation with QoL, −negative correlation with a subscale of QoL only, 0 no significant correlation with QoL*.

In cohorts of patients with acromegaly in remission only (11 studies), the general factor depression scores (23) was found to have a negative association with QoL. The disease-specific intervention previous radiotherapy (23, 24, 62, 65) had a negative effect on QoL subscales, whereas follow-up duration (23) had a significant negative effect on QoL subscales. No significant association was found for hypopituitarism (23, 62, 65) and remission duration (62, 63, 65) (see Table 6). Other predictors were either not reported, or no consensus was reached between the respective articles, such as the demographic factors age and gender, the biochemical parameters GH, IGF1, or biochemical control, and the duration of either disease, remission, or follow-up.

**Table 6:** **Factors influencing QoL in patients with acromegaly in remission (cross-sectional)**.

Reference	General factors			Disease-specific factors								
	Age	Female gender	Depression	Biochemical control	IGF1	Hypopituitarism	GH	Radiotherapy	Disease duration	Remission duration	Followup duration	Change in IGF1
Biermasz et al. (23)	−	0	−−		0	0	+	−	0	−	−	
Biermasz et al. (24)	−−						+	−	−−			
Bonapart et al. (25)					0		−−					
Hua et al. (9)				0								
Matta et al. (47)				+								
Neggers et al. (50)							0					0
Sardella et al. (58)				−					0			
van der Klaauw et al. (62)	+/−	−−				0		−		0		
Vandeva et al. (63)	−−									0		
Varewijck et al. (64)					−							
Wassenaar et al. (65)	+/−				0	0	0^a^	−	0	0		

*++positive correlation with QoL, +positive correlation with a subscale of QoL only, −− negative correlation with QoL, −negative correlation with a subscale of QoL only, 0 no significant correlation with QoL*.

*^a^Nadir GH*.

### Results from Intervention Studies

Three intervention studies demonstrated a significant positive effect of lanreotide autogel treatment on QoL of naïve patients with acromegaly (28, 29, 44), two of those detail the same cohort. Pegvisomant addition to somatostatin receptor ligands also demonstrated to have significant positive effects, both in a cohort biochemically well-controlled by somatostatin analogs (50) and with suboptimal control (61) (see Table 7). A cohort in which patients using octreotide LAR for at least 3 months demonstrated no effect of the injection interval on QoL (22). No significant effect was found for the interventions pegvisomant vs. octreotide-LAR, a study in which treatment-naïve patients were randomized between either pegvisomant or octreotide-LAR with QoL as a secondary outcome (37), physical activity (38), a crossover trial with patients switching to either pasireotide- or octreotide LAR (26) and somatostatin analogs vs. somatostatin analogs plus pegvisomant, a study in which patients controlled on somatostatin analogs were randomized to either continuation of treatment or co-treatment with pegvisomant (45) (see Table 7). Other predictors were either not reported, or no consensus was reached between the respective articles, such as surgery or octreotide treatment.

**Table 7:** **Interventions influencing QoL in patients with acromegaly (intervention studies)**.

Reference	Factor	Study characteristics	Therapy effect (i.e., QoL change after therapy)
Biermasz et al. (22)	Octreotide LAR interval injections	Patients uncontrolled on octreotide LAR (*n* = 14) receive 8 weeks washout followed by 6-week interval injections during 36 weeks	0

Bronstein et al. (26)	Crossover pasireotide LAR vs. octreotide LAR	Patients without biochemical control after 1 year of somatostatin analogs switched from either pasireotide LAR or octreotide LAR (follow-up 12 months after crossover)	0

Caron et al. (28)	Lanreotide autogel	Treatment-naïve patients (*n* = 90) with macroadenomas received lanreotide autogel during every 28 days for 48 weeks	++

Caron et al. (29)	Lanreotide autogel	Treatment-naïve patients (*n* = 90) with macroadenomas received lanreotide autogel during every 28 days for 48 weeks	++

Chin et al. (32)	Octreotide-LAR	Newly diagnosed patients (*n* = 58) were prescribed octreotide-LAR for 24 weeks	+

Fujio et al. (35)	Pituitary surgery	Newly diagnosed patients (*n* = 41) who achieved biochemical control after surgery were included	+

Ghigo et al. (37)	Pegvisomant vs. octreotide LAR	Medical-treatment and radiotherapy-naïve patients (*n* = 113), randomization between 4 weeks pegvisomant or octreotide LAR, followed by 48 weeks octreotide	0

Hatipoglu et al. (38)	Physical activity	Mixed cohort of patients (*n* = 20) exercised 3 days a week for 3 months. NB response rate <10%	0

Karaca et al. (40)	Octreotide-LAR	Treatment-näive patients (*n* = 22) were randomized to either octreotide LAR or pituitary surgery (follow-up 12 months)	0
	Pituitary surgery		0

Lombardi et al. (44)	Lanreotide autogel	Uncontrolled patients (*n* = 51) received autogel injections every 6–8 weeks (dose titration) for 48–52 weeks	++

Madsen et al. (45)	Somatostatin analogs vs. somatostatin analogs + pegvisomant	Patients controlled on somatostatin analogs (*n* = 18) randomized to unchanged continuation of somatostatin analogs or cotreatment with pegvisomant during 24 weeks	0

Mangupli et al. (46)	Octreotide-LAR	Retrospective observational study, patients (*n* = 28) on octreotide-LAR were followed for 4 years	++

Milian et al. (48)	Pituitary surgery	Patients selected for operative treatment (*n* = 93) were tested preoperatively and 3–12 months after surgery. No information on additional medical treatment	++

Neggers et al. (50)	Pegvisomant	Placebo-controlled crossover study (*n* = 20): patients controlled on somatostatin analogs receive addition of pegvisomant during long-acting SA-treatment in controlled patients during 36 weeks (2 × 16 weeks, 4 weeks washout)	++
	GH/change in IGF1 after pegvisomant addition		0

Trainer et al. (61)	Pegvisomant	Patients uncontrolled on octreotide-LAR (*n* = 27) randomized to pegvisomant monotherapy or addition of pegvisomant to octreotide-LAR	++
	Pegvisomant + long-acting octreotide		++

*++positive correlation with QoL, +positive correlation with a subscale of QoL only, −− negative correlation with QoL, −negative correlation with a subscale of QoL only, 0 no significant correlation with QoL*.

### Results from Longitudinal Studies

Longitudinal, defined as multiple-time point observational studies (nine studies), indicated that higher GH levels (25) as well as depression (36) had a significant negative impact on QoL during follow-up in patients with acromegaly. A reduction in IGF-1 significantly improved QoL (12); hypopituitarism had no significant effect on QoL in follow-up (62, 66). No significant contribution to QoL was found for the factor IGF-1 (25), disease duration (58), duration of remission (62), and education (66) (see Table 8). Other predictors were either not reported, or no consensus was reached between the respective articles.

**Table 8:** **Factors influencing QoL in patients with acromegaly (longitudinal studies^a^)**.

Reference	General factors				Disease-specific factors							
	Age	Female gender	Depression	Education	Hypopituitarism	GH	Biochemical control	Radiotherapy	Disease duration	Remission duration	IGF1	Change in IGF1
Bonapart et al. (25)						−−					0	
Caron et al. (29)							0					
Chin et al. (32)							−					
Fujio et al. (35)	−	0					0				0	
Geraedts et al. (36)			−−				0					
Paisley et al. (12)								0				−−
Sardella et al. (58)							−		0			
van der Klaauw et al. (62)	+/−	−−			0			−		0		
Webb et al. (66)	0	−−		0	0							

*++positive correlation with QoL, +positive correlation with a subscale of QoL only, −−negative correlation with QoL, −negative correlation with a subscale of QoL only, 0 no significant correlation with QoL*.

*^a^Longitudinal defined as: repeated measurements, observational*.

## Discussion

In the present systematic review that included 51 studies, we observed only a limited amount of randomized controlled trials with QoL as an endpoint. Only six randomized trials have been listed (37, 40, 44, 45, 50, 61), with only three of these investigating baseline data of treatment-naïve patients (37, 40, 44). Several risk factors that have shown a significant effect on QoL in cross-sectional observational studies have not been studied in a longitudinal design, such as depression scores and BMI. Studies investigating treatment for either depression or BMI (with the exception of one study that studied physical therapy but not formally targeted BMI) are absent. There are very few studies that assess long-term QoL patients with acromegaly throughout different phases of the disease; current studies do not properly reflect the transition a patient makes from active to controlled disease.

The general factors depression scores and BMI had a significant negative impact on QoL in a number of studies, in cohorts comprising both active and non-active patients. Intriguingly, the disease-specific factor hypopituitarism had no significant association with QoL in patients with acromegaly.

The association of depression scores with reduced QoL may be self-explanatory; previously, we have reported a marked superiority of depression scores over other predictors of QoL (23, 36). Increased scores for psychopathology have been described to be prevalent in patients with acromegaly (16, 21, 62, 68). Whether this observation is caused by acromegaly *per se* or is the result of a chronic disease in general is not clear. The demonstrated consensus on the significance of depression scores in QoL in patients with acromegaly provides further circumstantial evidence that paying attention to- and treatment of psychopathological comorbidities (by either psychological and/or pharmaceutical approaches) may provide added value to the chronic care of patients with acromegaly. However, no results on the effect of psychotherapy on QoL in patients with acromegaly have been published, whereas it has been a well-established intervention in several other chronic illnesses.

Higher BMI is considered to be associated with reduced QoL both in the general population and in those with chronic disease (69, 70); therefore, it is not surprising that there is a consensus on the significance of BMI as a factor in patients with acromegaly as well. Moreover, Turgut et al. reported that a polymorphism of the GH receptor leading to greater sensitivity (mirroring GH excess) correlated with increased BMI, suggesting a role of GH in acquiring greater body mass (71), independent of acromegaly. Moreover, obesity is a common symptom in acromegaly as GH excess changes one’s body composition even after biochemical control (72), making it a clinically relevant factor to be targeted in order to improve QoL in acromegaly. Up to now, only one study was performed aiming at weight reduction by physical activity, and this study failed to show an improvement in QoL (change in weight was not reported). Further research is needed to evaluate optimal weight reduction strategies in acromegaly and also take into account mobility issues related to arthropathy.

Many comorbidities, such as diabetes mellitus, which are more prevalent in acromegaly, may also influence QoL in this population. Most published studies have too small numbers to evaluate these parameters.

Many studies have however corrected for the effect of hypopituitarism next to demographic variables without taking into account its individual effect. The observation that hypopituitarism was not associated with QoL in patients with acromegaly may therefore be biased, because hypopituitarism *per se* is well-known to be a relevant factor in studying health outcomes in other pituitary diseases. With that in mind, we excluded all studies that investigated cohorts with GHD after acromegaly. Second, treatment of other hormone deficiencies in GHD patients has been demonstrated to significantly ameliorate QoL which further substantiates our motivation to exclude this specific condition from the present study (73–76). Moreover, the degree of hypopituitarism may play an important role in its association with QoL. Fathalla et al. described that pan-hypopituitarism had a significant negative effect on QoL (34), however, as this was the only study that investigated the role of pan-hypopituitarism it was not included in the final table.

Remarkably, no consensus on the role of biochemical variables has been reached. GH was shown in one study to be positively associated with QoL in active acromegaly, whereas this association was disputed in other cross-sectional cohorts. Although normalization of GH and IGF-I levels are obviously an important goal for treatment of acromegaly, our results strongly suggests that QoL in acromegaly is a different entity in addition biochemical control *per se* and may warrant clinical attention transcending current criteria for remission.

Although it seems likely that biomedical treatment of acromegaly would improve its symptoms, including an improvement of QoL, convincing evidence for this is as yet missing. The interventions of treatment with pegvisomant and somatostatin receptor ligands, in particular lanreotide autogel (from cross-sectional studies) reported a significant positive impact on QoL, while general treatment of acromegaly (not otherwise specified) and the comparison of surgery to somatostatin analogs were not reported to be significantly associated with QoL from available cross-sectional data. Obviously, it should be expected that treatments of a similar class as lanreotide autogel would ameliorate QoL in an equally similar fashion. Interestingly, the results on the effect of surgery, long-since the gold standard for cure of acromegaly, are conflicting. This effect, however, has not been investigated in longitudinal trials, rendering evidence on the benefit of surgery in QoL therefore inconclusive. Recent studies show a trend toward a relative positive effect of surgery on QoL, this trend is not supported by cross-sectional studies which predominantly show no correlation between surgery and QoL. Intervention trials are the optimum study design for investigating treatment, further research in larger populations than the current three intervention studies should be conducted to verify whether surgery indeed has a beneficial effect on QoL as it obviously is the first line treatment to establisg remission and amelioration of acromegalic symptoms.

Therefore, large trials with sufficient follow-up data which specifically studies QoL (generic and disease-specific) as a primary long-term outcome for each of the most widely used treatment strategies, either medicinal or surgical, are urgently needed. In an era of treatment choices and increasing focus on the patient perspective knowledge of the effect of distinct treatment options and modalities on QoL from prospective studies is of paramount importance to enable individually tailored decisions based on evidence based medicine.

The relation between GH/IGF1 excess and QoL is complex due limitations inherent to our current understanding of acromegaly. First, the reflection of disease activity is not straightforward and many different parameters to reflect GH excess have been used, usually single time-point measurements rather than time-weighted average GH activity that may not reflect tissue exposure. In addition, a direct comparison between naïve active patients and treated/cured patients is often lacking, both in prospective studies and in cross-sectional studies. Given the fluctuations in GH levels throughout the day, as well as individual differences in set point and sensitivity, it is uncertain whether decisive evidence proving the role of GH levels in QoL will be found in future research (77, 78).

Considering QoL as a complex entity with at least three domains (biological, psychological, and social components) (79), there may be disparity between generic QoL scores and disease-specific/domain-specific scores. Because of limited numbers and the use of many different questionnaires (*n* = 12), we could not evaluate results for all questionnaires. Limitations of this review include the small size of the studies with heterogeneous cohorts (mean *N* = 53.47, range 10–291) and the limited amount of longitudinal studies and intervention studies.

A difficulty in comparing studies of different designs (cross-sectional observational vs. intervention vs. longitudinal studies) is exemplified by differences in baseline QoL, particularly with regard to the range in QoL between the studies. Assuming a large difference in effect size of individual factors, direct comparison between the respective studies limits quantitative analyses.

A remarkable limitation in interpreting the results on biochemical control is the timeframe after which biochemical control was achieved. Although we report the parameters studied in both active acromegaly and acromegaly in remission, the largest portion of the studies was conducted in heterogeneous cohorts, which includes a large variety of disease- and follow-up durations. Despite this being a good reflection of clinical practice-cohorts, it may be beneficial to study QoL in acromegaly over an extended period of time, from diagnosis to long-term follow-up.

In conclusion, this article provides a comprehensive overview of the available literature until January 2017 for associative factors and predictors of QoL in acromegaly. It provides systematic evidence for the significant role of depression scores and BMI, but does not provide further arguments to support the role of biochemical parameters such as hormonal normalization as well as hypopituitarism and the main therapeutic modalities for acromegaly. At present, only interventions with lanreotide autogel and pegvisomant have shown to consistently improve QoL, while the effect of other interventions is either unclear or not properly assessed prospectively. Future research should include prospective and longitudinal measurements of QoL and the patient perspective to be able to use QoL scores in clinical decision making. Finally, treatment of depressive symptoms and BMI-reducing strategies are promising targets for QoL improvement strategies.

## Author Contributions

VG, CS, and NB conceived the study; are responsible for the integrity of the study; VG and CA collected and analyzed the data. All authors critically reviewed various draft of the manuscript and approval was consensual by all authors for the final version.

## Conflict of Interest Statement

The authors declare that the research was conducted in the absence of any commercial or financial relationship that could be interpreted as a potential conflict of interest.
