# Supplementary material for: Predictors of Quality of Life in Acromegaly: No Consensus on Biochemical Parameters
Source: Front Endocrinol (Lausanne). 2017 Mar 3;8:40. doi: 10.3389/fendo.2017.00040 (PMC5334635; doi:10.3389/fendo.2017.00040)
Supplement: Supplementary file 1 [file Table_1.DOCX]

| Supplement 1 Quality assessment | | | | | |
| --- | --- | --- | --- | --- | --- |
|  |  | NOS (max. 9) | | | QoL quality assessment  (max. 10) |
| Author + year | Type of study | Selection (max. 4) | Comparability (max. 2) | Exposure (case  control) / Outcome (cohort) (max.3) |  |
| Anagnostis 2014 | Case-control | **** | ** | * | 7 |
| Biermasz 2003 | Cohort | *** | * | ** | 5 |
| Biermasz 2004 | Case-control | *** | ** | * | 10 |
| Biermasz 2005 | Cohort | *** | ** | * | 10 |
| Bonapart 2005 | Cohort | *** | ** | ** | 8 |
| Bronstein 2015 | Cohort | *** | * | ** | 3 |
| Cannavo 2011 | Case-control | *** | ** | ** | 6 |
| Caron 2011 | Cohort | *** | * | ** | 3 |
| Caron 2016 | Cohort | *** | * | ** | 4 |
| Celik, Hatipoglu 2013 | Cohort | ** | ** | * | 8 |
| Celik, Kadioglu 2013 | Cohort | ** | ** |  | 8 |
| Chin 2015 | Cohort | ** | ** | ** | 4 |
| Dantas 2013 | Cohort | *** | ** | * | 9 |
| Fathalla 2014 | Cohort | *** | * | * | 6 |
| Fujio 2016 | Cohort | *** | ** | ** | 7 |
| Geraedts 2015 | Cohort | *** | ** | * | 6 |
| Ghigo 2009 | Cohort | *** | * | ** | 4 |
| Hatipoglu 2014 | Case-control | *** | ** |  | 4 |
| Hatipoglu 2015 | Cohort | *** | ** | * | 3 |
| Hua 2006 | Cohort | *** | ** | ** | 10 |
| Karaca 2011 | Cohort | **** | ** | ** | 4 |
| Kauppinen 2006 | Cohort | *** | ** |  | 6 |
| Kepicoglu 2013 | Cohort | *** | ** | * | 8 |
| Leon-Carrion 2010 | Case-control | **** | ** | ** | 6 |
| Lombardi 2009 | Cohort | **** | * | ** | 4 |
| Madsen 2011 | Cohort | *** | * | * | 6 |
| Mangupli 2013 | Cohort | **** | ** | * | 6 |
| Matta 2008 | Cohort | **** | ** | * | 7 |
| Millian 2013 | Cohort | *** | ** | * | 10 |
| Miller 2008 | Cohort | ** | * | * | 9 |
| Neggers 2008 | Cohort | **** | ** | ** | 4 |
| Paisley 2007 | Cohort | **** | * | ** | 7 |
| Postma 2012 | Cohort | *** | ** | * | 9 |
| Psaras, Honegger 2011 | Case-control | *** | ** | * | 7 |
| Psaras, Millian 2011 | Cohort | *** | ** | * | 10 |
| Raappana 2012 | Cohort | *** | * | * | 7 |
| Roerink 2014 | Case-control | *** | * | * | 10 |
| Rowles 2005 | Cohort | *** | ** | * | 7 |
| Rubeck 2010 | Cohort | *** | * | * | 6 |
| Sardella 2010 | Cohort | *** | ** | ** | 6 |
| Schopohl 2011 | Cohort | *** | * | ** | 3 |
| Siegel 2013 | Cohort | *** | ** | * | 9 |
| T’Sjoen 2007 | Cohort | *** | ** |  | 3 |
| Trainer 2009 | Cohort | **** | * | ** | 6 |
| Trepp 2005 | Cohort | *** | ** | ** | 5 |
| Vd Klaauw 2008 | Cohort | **** | ** | ** | 10 |
| Vandeva 2015 | Cohort | *** | ** | **** | 7 |
| Varewijck 2014 | Cohort | * | ** | * | 7 |
| Wassenaar 2010 | Cohort | *** | ** | * | 10 |
| Webb 2006 | Case-control | ** | ** | * | 8 |
| Yoshida 2015 | Cohort | *** | ** | * | 8 |
